# Living Sample Viability Measurement Methods from Traditional Assays to Nanomotion

**DOI:** 10.3390/bios12070453

**Published:** 2022-06-24

**Authors:** Hamzah Al-madani, Hui Du, Junlie Yao, Hao Peng, Chenyang Yao, Bo Jiang, Aiguo Wu, Fang Yang

**Affiliations:** 1 Cixi Institute of Biomedical Engineering, International Cooperation Base of Biomedical Materials Technology and Application, Chinese Academy of Sciences (CAS), Key Laboratory of Magnetic Materials and Devices, Zhejiang Engineering Research Center for Biomedical Materials, Ningbo Institute of Materials Technology and Engineering, CAS, Ningbo 315201, China; hamzah@nimte.ac.cn (H.A.-m.); duhui@nimte.ac.cn (H.D.); yaojunlie@nimte.ac.cn (J.Y.); penghao@nimte.ac.cn (H.P.); yaochenyang@nimte.ac.cn (C.Y.); jiangbo@nimte.ac.cn (B.J.); 2 University of Chinese Academy of Sciences, Beijing 100049, China; 3 College of Materials Sciences and Opto-Electronic Technology, University of Chinese Academy of Sciences, Beijing 100049, China; 4 Advanced Energy Science and Technology Guangdong Laboratory, Huizhou 516000, China

**Keywords:** living sample viability measurement, atomic force microscopy, AFM oscillating sensor method, nanomotion

## Abstract

Living sample viability measurement is an extremely common process in medical, pharmaceutical, and biological fields, especially drug pharmacology and toxicology detection. Nowadays, there are a number of chemical, optical, and mechanical methods that have been developed in response to the growing demand for simple, rapid, accurate, and reliable real-time living sample viability assessment. In parallel, the development trend of viability measurement methods (VMMs) has increasingly shifted from traditional assays towards the innovative atomic force microscope (AFM) oscillating sensor method (referred to as nanomotion), which takes advantage of the adhesion of living samples to an oscillating surface. Herein, we provide a comprehensive review of the common VMMs, laying emphasis on their benefits and drawbacks, as well as evaluating the potential utility of VMMs. In addition, we discuss the nanomotion technique, focusing on its applications, sample attachment protocols, and result display methods. Furthermore, the challenges and future perspectives on nanomotion are commented on, mainly emphasizing scientific restrictions and development orientations.

## 1. Introduction

The development and evaluation of new drugs take several years of investigations on living samples to explore drug pharmacology and toxicology. Compared to in vivo investigations on living sample viability, in vitro investigations are easier to execute and duplicate, the experimental settings are easier to regulate, and they are less morally problematic and costly [1]. In the past few decades, biological, chemical, and physical methods have been used for the rapid and accurate measurement of in vitro living sample behavior [2]. Living sample viability is a measure of the ratio of dead samples to live samples within a sample population. Living sample viability assays are used to assess the general health of samples and to track their survival after treatment with chemical agents or drugs. It is often expressed as a percentage of the control sample [3]. As the central parameter of living samples, viability is mainly measured through single-plate experiments or high-throughput screening, namely, pharmaceutical compound injection and living sample reaction record and assessment [4]. Apparently, living sample viability measurement plays an important role in clarifying the effects of drugs on cell proliferation and cytotoxicity, thus significantly reflecting drug safety and efficacy. For instance, living sample viability measurement provides great opportunities for analyzing the physiological behavior of anticancer drugs, such as selective ingestion and lethality in cancer cells, as well as biosecurity in non-tumor cells [5].

Recently, various chemical, optical, and mechanical methods possessing high accuracy and sensitivity have been developed in response to the demand for living sample viability determination [6]. Obviously, diverse measurement methods have their own superiorities and deficiencies depending on the application. Choosing the appropriate measurement method considers not only the test time, procedures, and the number of samples but also the application, cell line type, and host type. For instance, despite the extreme complexity of interpreting the experimental results of metabolic activity measurements, they have achieved significant progress. Among numerous viability measurement methods (VMMs), the atomic force microscopey (AFM) oscillating sensor method (named nanomotion), taking advantage of the adhesion effect of living samples to an oscillating surface, has emerged as a rapid, quantitative, real-time monitoring technique in the last decade [7]. To some extent, sufficiently detailed understanding of nanomotion strategy, from sampling attachment protocols to results display, will help achieve more reliable and repeatable living sample viability measurement.

This review aims to address the development of VMMs from traditional assays to nanomotion and to shed light on the novelty and practicability of nanomotion VMMs. Above all, various common VMMs are discussed and summarized in order to assess the potential areas of future development by discussing their most significant advantages and drawbacks. The use of nanomotion for monitoring living sample viability is discussed extensively through a comprehensive literature survey which summarizes the applications, the methods of sample adhesion on the microcantilever, and result display methods, and concludes with a consideration of the challenges and deficiencies that need to be addressed in the future. Finally, we hope that the review can promote the development of VMMs and present a promising innovative direction.

## 2. Living Sample Viability Measurement Methods

**Figure 1:**
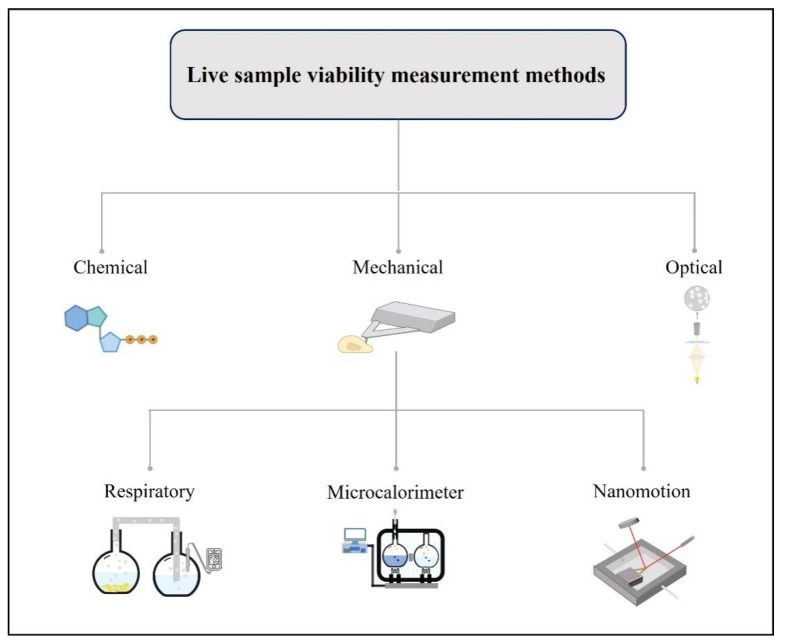
Viability measurement methods are classified according to the equipment or materials used in the measurement process, such as chemical viability assays and optical or mechanical methods.

Generally, In previous reviews, according to the measurement principle or measurement procedures, VMMs have been classified in previous reviews as direct or indirect, labelled or label-free, and endpoint or real-time [8]. While in this review, VMMs are classified according to the equipment or materials used in the measurement process. VMMs are classified into chemical, optical, and mechanical measurement methods, as shown in Figure 1.

### 2.1. Chemical Viability Assays

**Figure 2:**
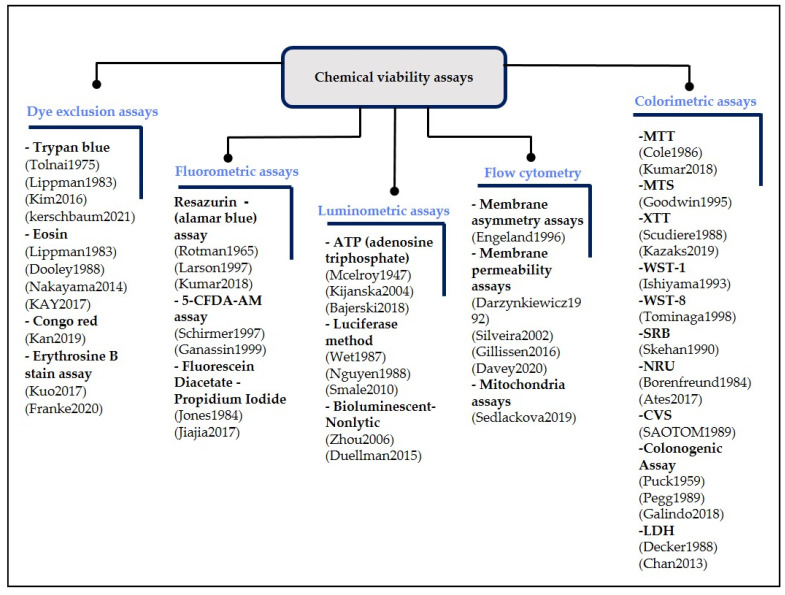
The broad classification of chemical viability assays and the various techniques they involve.

Chemical viability assays work according to a common principle—the injection of living samples with one or more compounds. For instance, an anticancer drug’s effectiveness or toxicity is evaluated through living sample interaction with drug’s compound(s) [4]. Therefore, chemical assay identification and design depend on the drug’s nature and vary according to the biomarkers used. The biomarker can be the outer surface of the sample membrane, nuclear size, or a metabolic process, such as the integrity of the membrane, adenosine triphosphate (ATP), the cellular esterases, enzyme function, and permeability. Chemical viability assays are mainly labelled, endpoint, and multi-sample methods. In general, chemical viability assays have several advantages. They are easy to perform, inexpensive, and rapid. They can be used to measure suspended or adherent samples and do not require complex techniques [1,9,10]. Figure 2 illustrates the wide classification of chemical viability assays and the various techniques they comprise.

Chemical viability assays are divided into five main categories: dye exclusion assays, fluorometric assays, luminometric assays, flow cytometry, and colorimetric assays. The principle of dye exclusion assays is based on the determination of membrane integrity. Dye exclusion assays determine the viability of suspension samples, with nonviable samples appearing in blue cytoplasm and living samples appearing in clear cytoplasm. Trypan blue [9,10,11,12,13,14] is a toxic assay for mammalian cells, and eosin [13,15,16,17], congo red [18], and erythrosine B stain assays [19,20] are nontoxic assays for mammalian cells. The principle of fluorometric assays is based on cellular esterases’ cleavage of a nonfluorescent compound into a fluorescent compound. Fluorometric assays are light-sensitive and are used to determine the viability of both suspensions and adherent samples.

Fluorometric assays include three methods: resazurin (alamarblue) assay, 5-CFDA-AM assay, and fluorescein diacetate-propidium iodide. In the resazurin (alamarblue) assay, a healthy sample undergoes a non-reversible enzymatic reaction that turns the resazurin or alamarblue into a pink color resorufin that spreads in the medium such that, by measuring color change, healthy samples can be calculated [21,22,23]. In the instance of the 5-CFDA-AM assay, the living sample’s enzymatic response transforms the assay into a fluorescent polar and impermeable solution that passes through healthy samples' cellular membranes. [24,25]. Propidium iodide, which interacts with the DNA of a dead sample, is combined with fluorescein diacetate, which is converted to fluorescein by esterase, to indicate apoptosis in the living sample. The combination of the two assays made it possible to measure the viability of living samples more accurately [26,27].

Luminometric assays include three methods: ATP (adenosine triphosphate), luciferase, and bioluminescent-nonlytic methods. The viability of living samples is determined in the ATP assay by using luminometers to assess intracellular ATP levels after the cells have been lysed to release intracellular ATP, which interacts with the luciferase enzyme [28,29,30]. The luciferase and bioluminescent-nonlytic methods are real-time viability measurement assays and can be used in continuous measurement applications [31,32,33]. In the luciferase method, cells are not dissolved to release ATP. Still, cells absorb the pro-substrate and turn it into a substrate that spreads in the medium. Bioluminescent-nonlytic assays include fluorescence and luminescent assays [10,34].

Flow cytometry includes three methods: membrane asymmetry assays, membrane permeability assays, and mitochondria assays. Membrane asymmetry assays are based on detecting changes in a cell membrane’s outer surface [35]. However, membrane permeability assays are based on detecting cell membrane integrity and permeability [36,37,38,39]. Mitochondria assays involve the detection of the membrane potential, mass, or membrane permeability of mitochondria [40].

Colorimetric assays are based on the determination of metabolic activity and can be applied to both suspensions and adherent living samples. Colorimetric assays include ten methods: MTT, MTS, XTT, WST-1, WST-8, SRB, NRU, CVS, colonogenic assay, and LDH. The MTT assay is converted into a colorful formazan by the active metabolism of viable samples, and the intensity of the colored formazan is proportional to the number of live samples [21,41]. Unlike the MTT assay, the MTS assay is directly soluble in the sample medium [42]. Through online data processing, the XTT assay enables the processing of a large number of samples with high accuracy and speed [43,44]. The WST-1 assay is water-soluble, eliminating the need for a separate formazan dissolving step [45,46]. The WST-8 assay was developed from WST-1 and has the advantage that it is less toxic and more sensitive than other types of colorimetric assays [46,47]. The SRB assay is designed to be more sensitive than the MTT assay based on its ability to bind essential amino acid residues to proteins and does not depend on metabolic activities in measuring the viability of living samples [48,49]. NRU diffuses easily across the plasma membrane and binds to anionic sites in the lysosome. The NRU principle is based on the ability of viable samples to bind the neutral red dye in the lysosomes [50,51]. The CVS assay principle is based on measuring sample adherence by coloring attached samples with CVS, a protein and DNA binding dye [52]. The principle behind the colonogenic assay, also known as the plating assay, is that live samples will generate colonies that are easy to observe for which the number of surviving samples can be easily estimated [53,54,55]. LDH is released into the extracellular space when the plasma membrane is disrupted, which can be a significant indicator of necrotic cells [56,57].

### 2.2. Optical Measurement Methods

An optical measurement strategy is a non-invasive way of measuring viability and monitoring the effect of drugs or toxicity [58] which provides an excellent opportunity to observe vital sample processes, including living sample functions and activities. The principle of most optical microscopy and imaging techniques is based on measuring diseased samples by observing their morphology, distribution, or interaction with specific antibodies.

In the case of living cells, the mitochondrial network is dynamic (fuse, divide, and move) [59] but the shape changes yielding vesicular punctiform mitochondria occur at the early stages of cell death [60] and the cell shape is fragmented into small punctuate and round structures that collapse to become isolated, expanded, and more numerous in the case of programmed cell death [61] and elongated or donut-shaped during autophagy [62]. In addition, during necrosis or apoptosis, when cells are under stress, this results in the occurrence of many irregular plasma membrane bulges inside the cells, the formation of many large vacuoles, and the detachment of tissue culture plates. Thus, monitoring the shape and position of cells by optical measurement methods can allow the rapid measurement of cell viability in real-time.

#### 2.2.1. Raman Spectroscopy

Raman spectroscopy (RS) is one of the most popular optical methods used to measure living sample viability [62,63]. Optical spectroscopy detects inelastic photon scattering caused by vibrational bonds in objects [64]. It is non-invasive and can be used to distinguish between healthy and dead samples. In addition, it is a rapid, label-free, real-time method that does not damage samples and works based on the sample’s interaction with electromagnetic radiation to provide chemical fingerprints [65]. Analyzing the RS images of living samples has made it possible to calculate samples viability as a percentage of dead or diseased samples compared to healthy samples. The RS images show the morphological changes of living samples, and the multivariate analysis processes these images using a software database. The application of multivariate analysis has enabled the classification of samples according to morphological changes in various subcellular organelles, such as nuclei, mitochondria, and cytoplasm. Therefore, samples can be classified according to their health status [66]. Using RS images and multivariate analysis recognized by custom software, cancer cells were compared with normal cells, providing an apparent discrepancy showing the different shapes of cancer cell components compared with those of normal cells [67]. By applying multivariate analysis to RS data, breast cancer cells were classified into responsive or nonresponsive as a function of drug dosage and type based on the evaluation of metabolic changes [64]. RS enables sample archiving and retesting for more precise therapy response assessment. The advantages of RS maps have been harnessed to quantify dynamic changes at the single-cell level in terms of sensitivity, for spatial and temporal resolution of multiplexed metabolic changes, and for quantitative analysis [64,68].

Recently, an automated platform approach for high-throughput screening RS was created to overcome human factor errors, reduce test time, and increase the number of samples under measurement [62,69]. The automatic development of RS algorithms involves analyzing vast quantities of data and the creation of a reliable and comprehensive database for machine learning [70] so as to increase the speed and reliability of testing. RS was recently combined with deuterium labeling, and the findings indicated that this novel RS detection technology might be used to identify cancer cells at the single-cell level [63]. RS’s challenges include the weak Raman signal [71] and light scattering [72], which reduce the method’s sensitivity. When interacting with a sample, scattered light can cause frequency deflection due to scattered photons. Spectrum pretreatments and scattered photon filtering can mitigate this effect and increase the quality of the process [72]. As new optical methods have emerged, such as flow imaging microscopy, holography, and on-chip, lensless video microscopy, which will be addressed later, the development of optical measurement methods has helped to overcome the method’s drawbacks.

#### 2.2.2. Flow Imaging Microscopy

**Figure 3:**
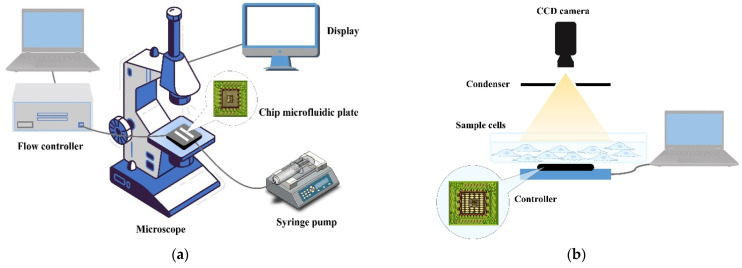
Optical measuring methods: (**a**) schematic of flow imaging microscopy (FIM) techniques; (**b**) digital holographic microscopy.

Flow imaging microscopy (FIM) is a rapid, label-free method used to determine living sample viability [73,74,75]. It is used to image the flow of fluids that contain vital components, such as human cells or protein particles. FIM captures the morphological changes of living samples and uses multivariate software to analyze FIM images to determine sample viability as a percentage of dead samples compared to healthy samples using the same working principle as Raman spectroscopy, though FIM takes sample images while the sample fluid is in continuous flow [75]. A specialized flow microscope is used for the measurement of living samples. In this system (schematically shown in Figure 3a), sequential bright digital images are captured when the sample passes through the flow cell. Living sample morphological information, number, size, and shape information are collected and then analyzed by software [76]. Flow imaging microscopy is a rapid and straightforward measurement method that reveals very subtle morphological changes in samples related to viability [75], such as mitochondria shape [60] and plasma membrane bulges [58]. The most important thing that distinguishes this technology from other optical microscopy techniques is its high throughput. FIM measures vital components individually one by one and calculates size distributions numerically using deep learning technology and a database generated by custom software [74].

Currently, the most significant limitation is the speed of data analysis [74]. Micro-flow imaging provided higher measurement precision, while FlowCAM showed higher-resolution images [75]. Recently, a study using a convolutional neural network for image analysis based on flow imaging microscopy techniques was carried out for a cell-based medicinal products test [73]. However, this required a long time for analysis using algorithms and the application of a machine-learning model to several databases [77]. FIM has proved to be a powerful tool for overcoming vital sample classification difficulties when used in conjunction with image-processing technologies and advanced machine-learning approaches. High classification efficiency improved a dataset by removing nonrepresentative photos logically and methodically. On the other hand, misclassification emphasizes how challenging it is to identify FIM images at a single level [78].

#### 2.2.3. Holography

Holography is a method for detecting living samples by observing morphological changes under stress or vibration resistance. Digital holographic microscopy provides a quantitative, contactless, non-destructive, and marker-free real-time monitoring method of living sample migration, adhesion, and dynamic change. It offers the possibility of measuring the efficacy of drugs in living samples [78,79]. Dead or diseased samples, for instance, will have a different intracellular structure from healthy samples. Changes in live sample structure parameters, such as volume, thickness, and intracellular composition, allow for the classification of living samples based on their health status and the calculation of viability percentages. Holography is a technique for measuring sample structure properties by scattering light after interacting with the sample. The scattering of light is affected by factors such as thickness, roundness, major axis, and intracellular composition [80]. Digital holographic microscopy is an optical microscopy technique that works on the interference between two waves, one from the sample and the other a reference wave from a charge-coupled device (CCD) digital camera, as shown in Figure 3b. In the context of the early diagnosis of cancer, a holographic microscope was used to distinguish between the morphology of cell tissues through a high-magnification optical technique that detects rapid changes resulting from mechanical or morphological changes. The method proved to be effective for cell thickness measurement in a culture medium [81]. It has been used to create high-resolution intensity images of a living sample and provide quantitative light phase and intensity information [80,82].

Due to its advantages, such as high efficiency, low cost, and flexibility to combine with other components, lens-free digital in-line holographic microscopy has become a valuable tool in the characterization and viability analysis of microbiological entities such as cancer cells [83]. Recently, a light-emitting diode has been used with the attachment of a pinhole structure as a practical light source. It enables direct observation of 3D bio-tissue without scanning and in the absence of noise caused by laser light [84,85].

#### 2.2.4. On-Chip, Lensless Video Microscopy Technology

On-chip, lensless video microscopy technology is a label-free, real-time, and non-destructive VMM technology with a field of view twice that of a conventional microscope [86,87,88,89,90,91,92]. This technology does not require optical elements, such as lenses, or mechanical elements, such as probes. The areas and dimensions of samples vary according to their health status. By capturing the shadows of living samples and analyzing these images, samples can be divided according to their validity. By analyzing the sample shadows captured in digital images, morphological changes in samples could be monitored in real-time. As a result of the shadow imaging provided by on-chip, lensless microscopy, living sample viability tests could be performed without the need for any labeling or reagents [87]. On-chip, lensless video microscopy technology monitors more than one living sample type simultaneously through the use of microfluidic channels [91]. Large-scale parallel automated imaging can be enabled for large sample populations with a set of microscopes on a chip with low cross-contamination risk [90]. Lens-free imaging allows for a high-throughput screen for living sample viability in situ at the point of use due to its imaging reduced footprint. Data can rarely be collected from such commonly used sites as incubators due to the inhibitory nature of collecting standard microscopic and spectroscopic equipment [93].

The combination of microfluidic microscopy and high pixel resolution eliminates the need for expensive lenses, light sources, and mechanical microscanning [89]. The iterative phase recovery algorithm demonstrated the ability to retrieve and evaluate sample information using image quality algorithms even without references. This was enhanced by using machine-learning techniques [94]. On-chip, lensless video microscopy technology can provide label-free, non-destructive, continuous monitoring in the fields of treatment drug tests and toxicity and proliferation measurements [87]. The on-chip imaging system allows the monitoring of entire populations of living samples while tracking the fate of individual living samples within the population [92]. The main disadvantage of these methods is possible phototoxicity, since the cells and tissues are usually not exposed to direct light during their life cycle. Therefore, the optical microscope process must be designed to minimize phototoxicity. This can be avoided by choosing an efficient microscope and a suitable detector [64].

### 2.3. Mechanical Measuring Methods

Several mechanical or physical techniques have been developed to quantify living sample viability. These methods are based on the principle of measuring or monitoring one of the vital activities of living samples. For any living organism, adhesion, respiration, proliferation, electrical charge, and thermogenesis process activities are vital signs of life. Several methods have been developed to measure these activities based on monitoring viability.

#### 2.3.1. Respiratory Measuring Methods

Respiratory activity is an essential metabolic activity. The ability to absorb and consume oxygen can be an important factor in indicating the ability of a living sample to survive. Monitoring the harm produced by chemical agents to the breathing activity of a living sample is used to determine the viability of living samples based on respiratory thermodynamic features. The percentage of dead samples relative to healthy samples can be calculated by comparing the oxygen absorbed by live samples with that absorbed by the controls [95]. Measuring the oxygen consumption of living samples requires closed containers isolated from ambient air [96]. Several techniques have been used to measure oxygen consumption, such as the Clark-type oxygen electrode [97] and electron paramagnetic resonance oximetry [98]. However, these methods have some disadvantages, including the difficulty of calibration, the risk of poisoning, the consumption of oxygen, and high costs, especially for large samples [96]. The optical oxygen sensor approach was utilized to avoid the limitations of the Clark-type oxygen electrode and electron paramagnetic resonance oximetry technologies. The optical oxygen sensor approach has been demonstrated to need periodic calibration, consume oxygen, and be sensitive to environmental conditions, such as temperature, pressure, flow, and salinity [99].

The measurement of oxygen consumption by living samples in tissue culture flasks has been carried out using an optical oxygen sensor [100]. The phosphorescence lifetime-based optical oxygen sensor is used to monitor viability response to chemical agents or toxics as a continuous, real-time, rapid, and high-throughput method [96]. While only optical contact between the probe and the fluorescent detector is involved, fluorescence-based oxygen sensors allow non-invasive detection through a clear container. Disposable sensors with fixed calibration are simple, inexpensive, and reliable, making them ideal for contactless microscale measurements. The device uses solid-state oxygen sensor inputs and a phosphorescence phase detector to detect the respiration patterns of living samples in a contactless manner. The sensor changes its phosphorescence lifetime in response to oxygen content, which does not need calibration and is monitored by a phase detector [96].

Oxygen-sensing microplates have been used to measure living sample viability using empirical correlations between fluorescence intensity and viability [101]. Algorithms have been set up to make assessments of the rate of oxygen consumed by living samples and to measure the theoretical correlation between fluorescence and viability [102]. Real-time and non-invasive measurements of oxygen uptake rate [103,104] and oxygen transfer rate [105] were directly correlated with living samples’ metabolic activity. Scanning electrochemical microscopy (SECM)-assisted oxygen consumption measurement, which changes with cell nanoscale height, has been used as a real-time method for measuring the viability of a single living sample [106].

**Figure 4:**
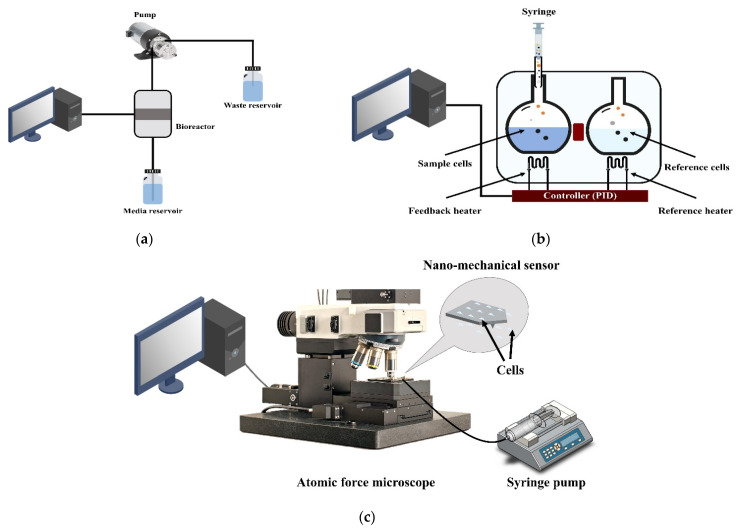
Mechanical measuring methods: (**a**) schematic of an online monitoring system based on respiration activity; (**b**) closed ampoule isothermal microcalorimetry; (**c**) nanomechanical oscillator sensor.

All metabolic processes in living organisms are heat-producing reactions [107]. Thus, heat flow indicates the number of metabolic reactions occurring in and the state of living samples [108]. Online oxygen monitoring is carried out by pumping the sample solution into a bioreactor containing a sensor sending signals. A computer translates these signals into data, as shown in Figure 4a. Recently, a method has been developed that combines cellular respiration measurement with measurement of living sample temperature changes and living sample proliferation rates directly by infrared thermal imaging, opening a promising avenue for the future of this technology [109].

Two central problems with the polydimethylsiloxane (PDMS) materials used today in the manufacture of microfluidic chips show the need to use thermal materials suitable for measuring the oxygen consumed by biological organisms. The high oxygen permeability of PMDS makes respiration and viable oxygen measurements difficult. Owing to its lipophilic nature, it has well-known absorption capacities for biomarkers and medications. Thermoplastic polymer materials with low oxygen permeability, such as polyethylene terephthalate (PET) or cyclic olefin copolymer (COC), are required as chip materials.

On the other hand, the process of manufacturing these thermoplastic polymer materials remains a significant challenge. The use of the manufacturing process for thermoplastic sensor integration is critical; it allows for repeatable measurements across a series of experiments. This technology, which can be easily integrated into existing thermoplastic microfluidic systems and enables living sample respiration monitoring, may pave the way for a more uniform and controlled means of monitoring culture conditions in cell-on-a-chip microfluidic systems [95].

#### 2.3.2. Microcalorimeter Measurement Methods

A microcalorimeter is an instrument designed for measuring the heat produced by microorganisms in closed bioreactors. By measuring the resulting heat employing a microcalorimeter, it is possible to directly monitor living organisms by comparing them with control samples. The percentage of dead samples relative to healthy samples can be calculated by comparing the heat energy produced by live samples to that produced by the controls [110]. Microcalorimetry can provide a continuous, direct real-time measurement of the activities of cellular components [107,110]. The heat energy vs. time curve is a complex construct used to measure a specific metabolic process. Still, it represents an ideal way to indicate total vital activities and living sample fate. Accurate microcalorimetric measurements are made using an isothermal calorimeter, where the measurement is carried out under constant temperature conditions. Many diverse studies in the literature refer to the use of isothermal calorimeters to measure the effect of pharmacokinetics on cells, microorganisms, and tissues, especially anticancer drugs [107,108,111,112]. Three parameters related to a living sample’s respiratory system have been used to gauge viability: respiratory intensity, proliferation rate, and normal sample heat. [109].

Closed ampoule isothermal microcalorimetry has been used to evaluate the vital activities of samples in continuous real-time monitoring. In closed ampoule isothermal microcalorimetry, the heat flow between a sample and a heat sink is measured and compared to the heat flow between a reference sample and the heat sink. The measurement is carried out isothermally. The heat flux is recorded as an electrical signal after the calibration [111], as shown in Figure 4b.

The indirect measurements of heat energy produced by living samples include non-contact temperature mapping by temperature-sensing methods. However, besides the complexity of results interpretation, accuracy is affected either by radiation absorption or the limitation of temperature resolutions. In the case of direct methods, the temperature change inside living samples can be measured by nanoscale thermal probing. However, this can cause additional heat production due to the stress response of sample rupture. The limited accuracy of the sensor’s energy resolution does not reach the level of single-sample temperature, which is at a level of pW. Therefore, most measurements are made by calculating the average heat generated by colonies of living samples, making determinations based on the calorimetry technique challenging.

The calorimeter principle makes it impossible to set up a sample development environment for an extended time. As a result, calorimeters are unsuitable for research in which living sample viability must be maintained for an extended period of time [113]. The most severe limitations of power resolution are thermal noise and noise created by microcalorimeter sensors. Thermal noise can be decreased by preventing heat exchange with excellent thermal isolation. Differential calorimetry is also an excellent method for removing interference. Furthermore, speedy systems are required for fast biological processes by reducing microcalorimeter chamber size. Moreover, developments in hardware, including data interpretation tools, will make microcalorimetry for living samples a standard tool [114].

Despite the literature indicating the possibility of using this method to measure cellular activities, only a few laboratories use microcalorimeters to measure vital activities. The reason for this may be high costs, which, according to the producers, are due to limited production levels. Still, perhaps by using accurate thermal electronic sensors, the demand for this technology will increase and costs will decrease [108].

#### 2.3.3. Micro-Nanomechanical Oscillator Sensors

The path of research on (bio)sensors has recently turned, with great interest, to micro-nanomechanical systems as precise measurement and monitoring systems. Micro-nanomechanical oscillator sensors appear as experimental real-time measurement techniques. They have enabled the exploration of biological, mechanical, and chemical properties in vital samples and the testing of molecular interactions, biological activities, and dynamic properties at the level of a single molecule and the temporal changes in these properties [115].

Cancer-marker detection and anticancer drug testing are increasingly needed for rapid, real-time, and high-sensitivity techniques. Micro-nanomechanical oscillator sensor techniques may provide the desired alternative [116]. Micro-nanomechanical oscillators are characterized as label-free biosensors. The sample does not require any previous treatment with colorimetric or fluorescent dyes. The other advantage of this technique is that it does not require as much time as colorimetric or fluorescent dye processes. Furthermore, it is possible to measure a single particle or a tiny sensing area of a sample [117]. On the other hand, the accurate measurement of a single living sample in the range of a few nanometers requires direct mechanical contact of the microscale cantilever with a single living sample [118].

Single-living sample measurement is one of the most important advantages of micro-nanomechanical oscillator sensors. Measurement experiments with a sample population can only give an average measurement. This neglects the individual differences between single samples and considers them as homogeneous. A better understanding of biological processes is achieved by considering the heterogeneity of samples, especially in toxicological and anticancer drug tests [119]. Micro-nanomechanical oscillator sensors are susceptible to minimal deflections (at nanometric scale) caused by very small forces (piconewtons). They can be used in low quantitative measurements and in parallel format [120,121].

Micro-nanomechanical oscillator sensors are vibrating mechanical structures that are often cantilevers. This microscale cantilever vibrates as an oscillating mass sensor to which the vital part adheres. The sensitivity of the vibration frequency or the oscillation amplitude depends on the mass of particles adhered to the cantilever. In oscillating mass sensors, either molecular receptors (e.g., protein) stick on the surface of the microscale cantilever or the living sample (e.g., a cell) adhere to the surface of the microscale cantilever.

Antibodies or other molecular receptors stick to the microscale cantilever surface, which is vibration movement-controlled. By moving it towards the sample, molecular recognition occurs between the sample target molecules and the sensor’s molecular receptors. This leads to mechanical, optical, or electrical interactions through which vital processes can be monitored [116,119,120,121,122,123,124].

Micro-nanomechanical oscillator sensors have been used to measure the interactions of surface receptors in vital samples. The chemical reactions of these receptors appear in the form of surface stress that can be traced and measured through micro-nanomechanical oscillator sensors. Surface stress caused by the receptor or the ligand causes micro-nanomechanical sensor deflection and changes in oscillation amplitude [123]. Some studies have used mechanical sensors for the dynamic examination of living cells [117,125].

Other studies have used mechanical sensors to monitor the activities of vital samples, such as cells or bacteria, including viability [120,126,127,128]. Understanding the mechanisms of the measurement process in the oscillation microscale cantilever is important for obtaining high measurement accuracy. Measurement accuracy depends on considering several factors when designing the measurement process. Factors include the viscosity of the oscillating medium, the adsorbed samples, the cantilever thickness compared to the thickness of the adsorbed samples, the adsorbed samples’ locations on the cantilever and their distance from the cantilever clamping region, and the mechanical properties of the cantilever material [117].

Changing the health status of living samples alters some mechanical properties, such as weight and stiffness, and some biological properties, such as adhesion [129,130,131]. The oscillation amplitude of the micro-nanomechanical sensor varies with the alteration of adhering living sample mechanical properties and this is the principle behind measuring viability [128]. Healthy living samples may undergo death when their adhesion to the surface or the extracellular matrix (ECM) is lost. Undoubtedly, it is notable that cell death is accompanied by the loss of adhesion bonds with the surface or ECM [127]. The significance of this stems from adhesion being essential for viability. As a result, many researchers have developed theories to measure adhesion using nano-micromechanical sensors to monitor viability and toxicity.

One piece of advanced nanomechanical oscillator sensor technology that enables real-time, direct, label-free measurement is the atomic force microscopey (AFM). Living samples are connected to an AFM cantilever and placed in a test chamber. The vibrations of the cantilever are tracked over time. An optical transduction system detects and records cantilever dynamic oscillation deflections by reflecting a laser beam from the oscillation cantilever to a detector, as seen in Figure 4c.

AFM has been used in many measurement techniques and can be described as having three main components: an imaging mode, a force spectroscopy mode, and an oscillating sensor mode. AFM can directly image single membrane proteins and living samples at nanometer resolution in a buffer solution—a crucial advantage over other microscopy techniques. Real-time AFM imaging of single living samples can provide novel insights into dynamic processes [132]. The AFM force spectroscopy mode is, among others, used to measure the interactions of biological systems. The AFM’s cantilever tip applies force to the living sample. This force may be a tensile force, a pressure force, or a shear force. AFM can be used to investigate the mechanical properties of microbiological systems ranging from tissues to nucleic acids [133]. Sample shape changes during its life cycle due to mechanical properties, such as internal and external forces [134]. AFM is capable of applying pN to nN forces to microscale indentors, allowing surface tension and tissue stiffness measurements [135]. In the detachment event, this technique directly measures a single sample’s detachment force from the surface by applying vertical pressure force [134] or shear force [136,137,138,139]. This technique provides a label-free, rapid, and quantitative method to take measurements at the single-cell level [136]. By recording the deflection of the cantilever, the adhesion force can be obtained according to Hooke’s law [140].

## 3. The AFM Oscillating Sensor Mode (Nanomotion)

### 3.1. Nanomotion Introduction

In measuring adhesion by single-cell force spectroscopy, a living sample is attached by force, which is contrary to the natural adhesion phenomenon. Natural living sample adhesion takes a longer time. It occurs to a lesser degree due to natural factors, such as gravity or the self-propulsion of the living sample [141]. Living sample adhesion is also measured in a detachment event that occurs after adhesion directly. However, adhesion, the number of bonds and the area of contact typically increase with time [142]. This causes the measurement process to be unrealistic and affects the accuracy of the results obtained. This adhesion fact motivated the creation of novel strategies that enable AFM to be used to evaluate adhesion in real-time without putting the living sample under stress by rapidly stretching it or pushing it to detach through cliffs. The AFM oscillating sensor modes are innovative methods that have recently been used to measure the adhesion of living samples, especially in measuring cell viability and the effects of chemical agents, such as drugs and toxic substances [143,144].

On the other hand, the traditional AFM methods provide end-state visualizations of sample fates as effects of chemical agents. They do not show the instantaneous effect of chemical agents on living samples. The real-time quality of nanomotion gives another additional advantage over AFM single-cell force spectroscopy methods.

**Figure 5:**
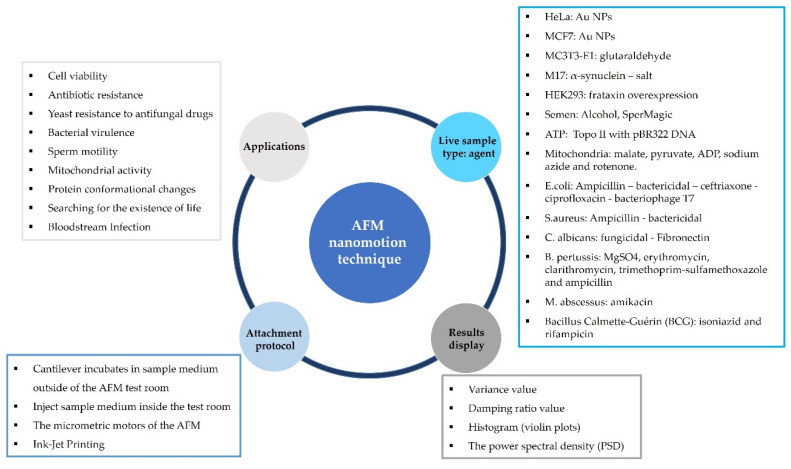
The AFM nanomotion technique. Different applications of nanomotion technology have been used for several types of living samples with different chemical agents. Different protocols were used to adhere the sample to the cantilever surface. Different display methods were used to present the results.

The operating principle of this method is to take advantage of the high sensitivity of the AFM cantilever. Its high flexibility allows high sensitivity to the nanomotion resulting from the change in mass due to the adhesion of nanoparticles to the cantilever surface. The fundamental principle behind these biological oscillating frequencies is the measurement of the change in frequency response caused by the additional load of biomolecular mass attached to the cantilever surface. In general, AFM oscillating cantilevers measure cantilever deflection or frequency response changes caused by a mass of adhered biomolecules [92]. As a result, AFM oscillating cantilevers are being explored as sensitive mass detectors for biological tracking systems. Figure 5 illustrates a literature survey of AFM nonomotion techniques.

The deflection of a cantilever is proportional to the force. It results from the interaction of the cantilever with the sample according to Hooke’s law [127]:

Δz = k_f_^−1^ F (1)

where Δz is the deflection, k_f_ is the spring constant, and F is the acting force. In case a force is applied on a harmonical oscillation, the amplitude variation depends on the loading force, while the frequency remains constant, as shown in Equation (2) [127]:

(2) $$z\;=\frac{F\cdot x^{2}\left(3L-x\right)}{6\mathrm{EI}}=\frac{g\cdot x^{2}\left(3L-x\right)}{6\mathrm{EI}}\cdot m_{\mathrm{cell}}$$

where x is the position of cell mass m_cell_ on a cantilever of length L and g, E, and I are the gravity coefficient, Young’s modulus of the cantilever, and the moment of inertia of area, respectively.

Biomolecules are attached to the surface of an AFM cantilever that is implanted into a test chamber. The transformation of the cantilever oscillations over time is then tracked. An optical transduction system detects and records the dynamic oscillation frequencies of the cantilever via a laser beam reflected from the oscillating cantilever to a detector. This system’s time resolution and sensitivity are ideally suited to studying living organisms at the nanoscale [145,146]. The AFM oscillating cantilever method provides a simple, highly weight-sensitive possibility for direct, real-time and single- or multi-cell measurements with high accuracy. When using the cantilever to evaluate cancer cells, an increase in the cantilever’s deflection and a change in oscillation frequency indicates when cancer cells are adhered to the cantilever’s surface.

The AFM oscillating sensor mode or AFM nanomotion makes for easier procedures than AFM single-cell force spectroscopy techniques. Still, the method neglects the heterogeneity of cells, though this is an advantage, since we are rarely interested in single-cell behavior but instead in statistical values. The AFM single-cell force spectroscopy methods that measure a single cell’s adhesion do not yield high productivity in the measurement of anticancer drugs and cell viability. Furthermore, because of the irregular shape of single cells, theoretical models cannot describe the resulting changes in their shapes due to applying compressive or shear forces to the cells, making methods for calculating cell adhesion strength by surface stress very complex. The shape of a cell strongly influences the applied force, making the calculation of the exact adhesion force difficult.

This novel method serves as a new technique for monitoring cell viability by measuring cell adhesion and thereby as a new technique for testing drug efficacy and toxicity. The changes in the values of the cantilever’s deflection and the frequency of vibration appear as a result of changes in cell state and cell detachment from the cantilever’s surface as a result of death. As a result, this method could be used to assess the efficacy of drugs and toxins. It has provided many advantages over traditional methods for measuring cell viability, including direct, real-time, and label-free measurement in tens of minutes. In contrast, traditional methods take days or even weeks. This technique complements traditional VMMs. It may be promising for the long-term development of cell viability measurement. Unlike optical and electrical measurement methods, this test can give a quick and reliable direct measurement of the viability of biological organisms, even if they are not characterized [127,147].

### 3.2. Nanomotion Application

The AFM oscillating cantilevers were initially used as sensors for detecting the presence of bacteria and germs by measuring the effect of bacteria and germs attached to their surfaces in air or liquids. The presence of bacteria or germs was indicated by a change in a cantilever’s vibration amplitude and frequency [117,143].

AFM oscillating cantilevers have been used to study the biological activities of bacteria as well as the effects of antibiotics and medicines on them, as shown in more detail in Table 1. The effect of antibiotics on bacteria was detected by changing the vibrations of the AFM oscillating cantilever, as living bacteria produced a larger cantilever deflection compared to antibiotic-treated bacteria [126]. A physical model has been developed to approximate the sum of the spectral frequencies caused by different amplitudes and the frequencies caused by adherent bacteria at various locations on a cantilever [147]. A measuring device based on the AFM oscillating cantilever principle was developed to measure minimal inhibitory and bactericidal concentrations and bacterial metabolic activities [148].

In the case of bloodstream infection, an innovative, rapid early detection of infecting microorganisms was obtained along with an accurate determination of their antibiotic susceptibility using the AFM oscillating cantilever [149]. Nanomotion was employed to detect sperm motility caused by exposed chemical agents. Living sperm produced less deflection with inhibitory chemicals and more deflection after treatment with stimulatory chemicals [150]. One of the recent studies used the AFM oscillating cantilever technique in laboratory experiments that could be used in the search for living organisms in space. The researchers compared the sensitivity of the frequencies and the amplitudes of cantilever vibration resulting from the adhesion of various biological organisms, such as bacteria, yeasts, animal cells, plant cells, and human cells. The study found that when living organisms died, the cantilever’s oscillation amplitude decreased [92,120].

The AFM oscillating cantilever technique has been used to monitor cell viability [120,127,144,151,152] by measuring cantilever deflection change due to cell detachment. The effect of anticancer drugs and toxins on a cell’s fate and metabolic activities could be successfully derived. The AFM oscillating cantilever technique was also applied to cellular organs, such as mitochondria [145] and ATP [153]. A difference in oscillation was found depending on the state of the mitochondria and their metabolic activity [145]. A group of researchers employed nanomotion to analyze the dynamics of enzymes in response to ligands such as ATP, and this has provided a novel way to investigate protein–ligand interactions [153]. 

Wu et al. examined the effect of paclitaxel on the MCF-7 breast cancer cell line. The cells settled down onto the microcantilever due to gravity. The attached cells were cultured and incubated for 4 h at 37 °C in a humidified atmosphere of 5% CO_2_ until they adhered to the cantilever. The positions of the adhered cells on the cantilever surface were controlled without cells on the free end, which were used to reflect the laser beam [143]. Without controlling the locations of the adherent cells on the surface of the cantilever, but in the same way as adhering cells and under the same conditions applied for incubating the cells, Ruggeri et al. studied the response of a neuron model system to monomeric and toxic amyloid aggregated species of α-syn using an M17 dopaminergic neuroblastoma cell line [152]. To assess dose-dependent toxicity and monitor cell viability by measuring cell adhesion, Yang et al. used an AFM microscope as an early cell-death marker [127]. Three different sizes and surface coatings of Au NPs were added to a HeLa immortal cell line and an MCF-7 breast cancer cell line to measure cell viability. Nanomotion was used to provide information on the cell metabolic changes caused by frataxin deficiency under oxidative stress conditions [151].

**Table 1:** Literature survey of AFM nonomotion viability measurement method.

Attachment Protocol	Results Display	Application	Cell Type	Time	Agent	Cantilever Type	Cantilever Functionalization	Ref.
Inject sample medium inside AFM test room	Variance value	Antibiotic resistance	*E. coli* and *S. aureus*	60–90 min	Ampicillin	DNP-10, Bruker	APTES (0.2%, 1.5 min)	[126]
Cantilever incubates in sample medium outside of the AFM test room	Variance value	Antibiotic resistance	*E. Coli*	2 h	Ampicillin	DNP-10, Bruker	Glutaraldehyde (0.5%, 7 min)	[154]
Cantilever incubates in sample medium outside of the AFM test room	Variance value; power spectral density	Protein conformational changes	Ligands, such as ATP	<10 min	Topo II enzymes with Pbr322 DNA (200 nm)	DNP-10, Bruker	APTES (0.1%, 1 min)	[153]
Cantilever incubates in sample medium outside of the AFM test room and Micrometric motors of the AFM (AFM single-cell force spectroscopy)	Variance value	Life-searching experiments on Earth and interplanetary missions	*E. coli*	>190 min	Bactericidal dose (10 μg/mL)	DNP-10, Bruker	Glutaraldehyde (0.5%, 7 min)	[120]
			*S. aureus*	>190 min	Bactericidal dose (2 μg/mL)		Glutaraldehyde (0.5%, 7 min)	
			*C. albicans*	>190 min	Fungicidal dose (20 μg/mL)		Glutaraldehyde (0.5%, 7 min)	
			MC3T3-E1	>190 min	5% glutaraldehyde		Fibronection (10 μg/mL, 15 min)	
			M17	>190 min	Salt concentration increasing		Poly-L-lysine (10%, 30 min)	
Cantilever incubates in sample medium outside of the AFM test room	Variance value	Cell viability	MCF7	7 h	Paclitaxel	DNP-10, Bruker	APTES (10%, 30 min)	[144]
Inject sample medium inside AFM test room	Damping value	Cell viability	Hela and MCF7	4–5 h	Au NPs	SNL-10, Bruker	-	[127]
Micrometric motors of the AFM (AFM single-cell force spectroscopy)	Variance value	Single-cell cytotoxicity assays	M17	7 h	Extracellular monomeric and amyloid α-synuclein species	DNP-10, Bruker	Poly-L-lysine (10%, 30 min)	[152]
Cantilever incubates in sample medium outside of the AFM test room	Variance value	Bloodstream infection	*E. coli*	90 min	Ceftriaxone, ciprofloxacin and ampicillin	NP-O10, Bruker	Glutaraldehyde (0.5%, 7 min)	[149]
Cantilever incubates in sample medium outside of the AFM test room	Variance value	Mitochondrial activity detected	Mitochondria- embryonic kidney cells	110 min	Malate, pyruvate, ADP, sodium azide, and rotenone	NP-O10, Bruker	Glutaraldehyde (5%, 10 min)	[145]
Inject sample medium inside AFM test room	Variance value	Sperm motility	Semen	-	Alcohol, spermagic	-	APTES (10%, 15 min)	[150]
Cantilever incubates in sample medium outside of the AFM test room	Variance value	Antibiotic resistance	*B. pertussis*	100 min	Erythromycin (Sigma- E6376); clarithromycin (Sigma -A3487), trimthoprim-sulfamethoxazole	-	Glutaraldehyde (0.5%, 10 min)	[148]
Cantilever incubates in sample medium outside of the AFM test room	Variance value	Antibiotic resistance	Bacillus Calmette-Guérin (BCG) and *M. abscessus*	200 min	BCG vs. Isoniazid and rifampicin *M. abscessus* vs. Amikacin	DNP-10, Bruker and SD-qp-CONT, NanoandMore	Glutaraldehyde (0.5%, 15 min)	[155]
The micrometric motors of the AFM (AFM single-cell force spectroscopy)	Variance value	Cell metabolic changes	HEK293	40 min	Frataxin overexpression	DNP-10, Bruker	Poly-D-lysine (20 μg/mL, 15 min)	[151]
Inject sample medium inside AFM test room	Variance value	Antibiotic resistance	*E. coli*	120 min	Bacteriophage T7	RC800PSA, Olympus	Poly-L-lysine (0.01%, 15 min)	[156]
Cantilever incubates in sample medium outside of the AFM test room	Variance value	Yeast resistance to antifungal drugs	*C. albicans*	>2 h	Fibronectin	Qp-CONT, nanoandmore	Con A (2 mg/mL, 30 min)	[157]
Cantilever incubates in sample medium outside of the AFM test room	Violin plots	Bacterial virulence	*B. pertussis*	5 min	Mgso4	SD-qp-CONT, nanoandmore	Poly-L-lysine (0.1%, 5 min)	[158]
Cantilever incubates in sample medium outside of the AFM test room	Variance value	Viability and susceptibility of microorganisms	*E. coli* and *S. aureus*	4 h	Ampicillin, glutaraldehyde	SD-qp-CONT, nanoandmore	Glutaraldehyde (0.5%, 10 min)	[159]

### 3.3. Attachment Protocol

**Figure 6:**
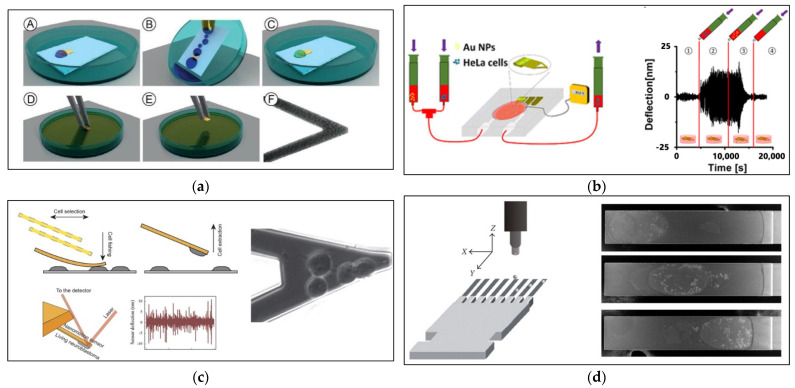
The AFM nanomotion technique attachment protocols: (**a**) cantilever incubated in sample medium outside the AFM test room, (A) functionalizing chemical is placed on the cantilever surface, B) the remaining chemical is washed using pure water then the cantilever allowed to dry, (C) sample is deposited on the cantilever surface, (D), (E) cantilever is immersed in and out of the culture medium to remove loosing samples, F) make sure attachment done with sufficient number of sample and no loosely attached samples, “reprinted with permission from Ref. [160]. 2018, École polytechnique fédérale de Lausanne”; (**b**) injection of sample medium inside the test room, (1) the cantilever vibrates at a specific frequency and the deflection is recorded over time, (2) samples are injected and allowed to adhere to the cantilever; as cells attach, deflection increases, (3) chemical agents are injected, and when cells start interacting with the agents, cells start to detach from the cantilever, causing the deflection decrease, (4) the cantilever is washed in preparation for the following measurement cycle, “reprinted with permission from Ref. [127]. 2017, Springer Nature”; (**c**) the micrometric motors of the AFM or the AFM single−cell force spectroscopy, “reprinted with permission from Ref. [152]. 2017, Springer Nature”; (**d**) ink−jet printing method, “reprinted with permission from Ref. [161]. 2012, Hindawi Publishing Corporation”.

The sample’s adhesion to the surface of the microcantilever is crucial to the success of the measurement method. The measurement process principle is based on the adhesion of the cell or the bacteria to the surface of the cantilever as an indication of its viability, with non-adhesion as a sign of death. Choosing the appropriate adhesion protocol depends on the sample’s nature, size, and concentration. There are four main factors to consider when choosing a protocol for sample immobilization on the cantilever surface: the process of adhesion should take place in an environment that preserves the live sample; maintenance of the environment under the same conditions during all stages of the process; the possibility of controlling the location and number of cells or bacteria on the cantilever surface; and the risk of contamination, sample death, or cantilever damage. Figure 6 is an illustration of four techniques used in sample immobilization.

The direct attachment method is the most commonly used, the easiest, and the least expensive method. The cantilever is incubated in the live sample medium outside the AFM test room; see Figure 6a. This method has been used to measure the viability of bacteria [148,149,151,153,154,155,157,158,159] and cells [120,144,145]. The method is carried out by placing a small amount of high-concentration sample medium directly over the cantilever and leaving it for a period of time until the sample settles on the cantilever. After that, the suspended samples are washed by dipping the cantilever in a medium. Then, the cantilever is transferred to the test room and mounted on the AFM. Before placing the sample medium on the cantilever, it is prepared by applying a quantity of certain functionalizing chemicals for a period of time; the cantilever is then washed with water and dried [160]. The drawbacks of this method include the different conditions under which the adhesion process takes place compared to the conditions of the chemical effect process, while the transfer of the cantilever out of the medium between the two stages affects the accuracy and reliability of the test. The adhesion process is carried out under different conditions of the chemical effect process and the inability to control the number of samples and their position above the cantilever’s surface, random sedimentation, and the possibility of contamination, sample death, or cantilever damage when handling and installing the cantilever are some of the additional disadvantages of this method.

To overcome the drawbacks of direct immobilization, a high concentration of live sample medium is injected inside the test room [126,127,150,157]. For this purpose, an injection system containing a cantilever was designed inside a fluid chamber inserted into the head of the AFM, as shown in Figure 6b. The AFM cantilever was housed in the thermostatically regulated and sealed test section with in-and-out liquid connections. Samples and chemical agents were collected in syringes. The cantilever oscillated with a constant amplitude at a certain frequency over time. The living samples were then injected and allowed to adhere to the cantilever for a period of time. Thus, the cantilever deflection increased with the increase of adherent living samples. When the cantilever had reached a stable state, the chemicals were injected into the test room. After a while, the effect on the living sample began, and the dead samples detached from the cantilever. In this case, the cantilever’s deflection decreased as the number of dead samples increased until it reached its original state after death and all the samples detached. The cantilever’s instantaneous deflection was controlled via feedback based on the detection of a camera installed on the microscope [127]. The living samples and chemical agents injected were implemented in the same test room and under the same conditions. Performing all steps under the same conditions enhances the accuracy and reliability of the test. There is no risk of contamination or death of samples due to the handling and installation of the cantilever with attached cells. However, the drawbacks of this method include random sedimentation, the requirement for a high sample concentration, and the inability to control the number of samples and their position above the cantilever’s surface.

AFM single-cell force spectroscopy (the micrometric motors of the AFM technique) [151] was used as a sample attachment protocol. The single force technique was originally used to measure the strength of single-cell adhesion. The change in cantilever deflection was measured as the change in the force needed to overcome the adhesion force of a cell (attached to the cantilever) to another cell, to a cell mass, or to a surface [162]. In preparation for the measurement process, a cell is attached to the surface of the cantilever by pressing it against it for a certain period of time, then lifting the cantilever and the cell attached to it; see Figure 6c. Sample immobilization in nanomotion is accomplished by aligning the functionalized cantilever above the single cell and then lowering it until it presses against the cell with a certain force for a certain length of time (5 nN, >3 min). The cantilever is then lifted with the cell attached to its lower surface. The process is repeated once to adhere a new cell in another place on the lower cantilever surface and repeated again depending on the required number of cells. As a result, this method can be used to detect the nanomotion behavior of a small number of cells while maintaining a high level of control over the number and location of samples [120,152]. The AFM single-cell force spectroscopy immobilization protocol is characterized by the possibility of conducting a nanomotion test for a single cell or multiple cells. The location and number of cells or bacteria can be controlled. The adhesion and chemical effect processes are carried out in the same test room and under the same conditions.

However, this technique is more complex and requires expensive equipment. A sample is limited by its size and by cantilever size. There is substantial potential for misinterpretation of data due to cell damage during the adhesion process [163]. Studies have demonstrated the effect of bacterial adhesion position distribution on the cantilever, and the effects increased when the adhesion positions were close to the cantilever’s free end [164]. The cantilever vibration due to the attached bacteria was caused not only by the mass effect but also by the bacterial cells’ stiffness. This directly affected the sensitivity of nanomotion technology. By comparing theoretical results with measurements in air and deionized water, the viscosity effect of the measuring medium was determined [117].

Ink-jet printing has been employed to immobilize samples on cantilevers, demonstrating its superiority as a method for nanomotion and real-time monitoring measures [161]. The ink-jet technique enables the choice of immobilized samples’ positions on cantilevers, which enables the study of the effects of sample location on cantilever nanomotion behavior; see Figure 6d. Controlling the location of samples allows for more flexibility in selecting optimal positions for reflection, resonance, and low noise [143]. The ink-jet printing immobilization process is monitored by a charge-coupled device (CCD) [163]. The ink-jet printing immobilization protocol is characterized by the fact that the location of cells or bacteria can be controlled. The adhesion and chemical effect processes are carried out in the same test room and under the same conditions. However, with this technique, it is not possible to control the number of cells or bacteria; it is also complex and requires expensive equipment. Table 2 provides a summary comparison of AFM nonomotion living sample attachment protocols.

### 3.4. Results Display

Many sensitive displacement sensors have been developed in order to read out and display the minute deflections of microcantilevers. Optical beam deflection, piezoelectric, and piezoresistive read-out techniques are the ones most commonly used [134]. The most popular read-out technique is optical beam deflection. For a single sensor, it is easy to execute and achieves angstrom resolution. A laser is focused on the cantilever free-end and reflected from it to be detected by a position-sensitive photodiode. A photodetector measures the displacement of the reflected laser beam due to cantilever deflection. The AFM controller receives data from the photodetector and transfers them to a PicoForce spectrometer for deflection recording. A multimeter records the data from the PicoForce spectrometer and then transfers it to the monitoring system [127]. The resulting data do not show static deflection but are presented on a time-dependent deflection chart. The dynamic deflection data collected with the cantilever are usually analyzed using homemade software. The deflection signal is represented as a continuous curve that monitored the cantilever’s deflection over time [126]. The amplitude variance of cantilever oscillations that appears in experimental results reflects the metabolic state of the biological samples and the effects of the chemical agents. Several methods were utilized to display deflection data so that the effects of chemical agents on samples could be compared clearly and productively.

**Figure 7:**
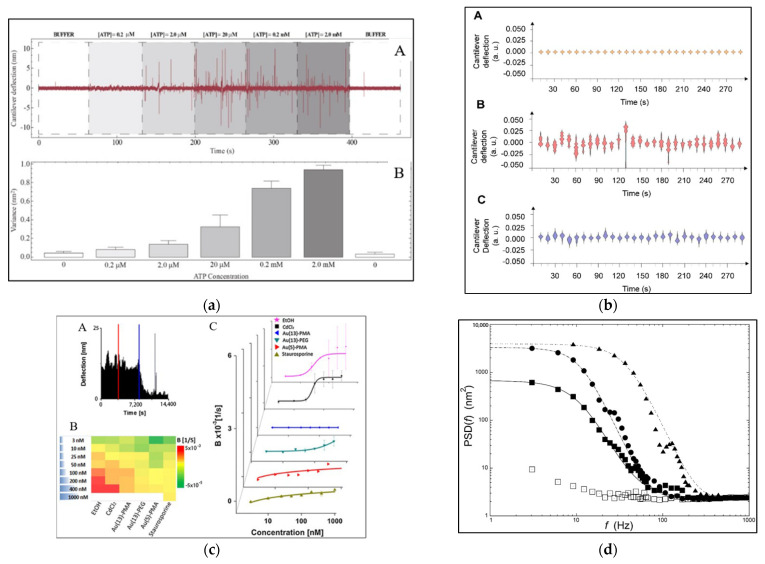
The AFM nanomotion technique results display methods: (**a**) the variance of the cantilever deflection result, (A) the cantilever deflections as a function of ATP concentration, (B) corresponding variance values, “reprinted with permission from Ref. [153]. 2014, Plos One”; (**b**) violin plot for 10 s chunk of the cantilever deflection result, (A) nanomotion cantilever violin plot without samples, (B) with virulent sample and (C) with avirulent sample, “reprinted with permission from Ref. [158]. 2021, MDPI”; (**c**) damping value (B value) of the cantilever deflection result, (A) cantilever oscillation deflection amplitude versus time, (B) heatmap of the damping constants, (C) damping constants B for different agents versus agents' concentration, “reprinted with permission from Ref. [127]. 2017, Springer Nature”; (**d**) the power spectral density (PSD) of the cantilever deflection result (black squares: 2.0 μM ATP concentration, black circles: 0.2 mM, black triangles: 2.0 mM and white squares: baseline), “reprinted with permission from Ref. [153]. 2014, Plos One”.

Many studies have used the variance of cantilever deflections to compare results from different experiments, as shown in Table 1. When the living samples adhered to the cantilever, the deflection variance increased, but after the injection of chemical agents and their interaction with the samples, the variance value reduced dramatically, indicating that the vital samples had died, as shown in Figure 7a. The following equation was used to determine the variance (Var) values that were utilized to quantify the deflection fluctuations (*z_i_*) [153]:

(3) $$\mathrm{Var}\;=\frac{1}{N-1}\overset{N}{\underset{i=1}{\sum }}{\left(z_{i}-z\right)}^{2}$$

As the number of samples adhering to the cantilever increases, the variance bars used to show the cantilever’s deflection increase. The variance decreases as the number of samples adhering to the cantilever decreases. If the number of samples on the cantilever remains constant, the cantilever oscillates with a steady variance [154].

Expressing the results by variance is an easy and practical method that enables clarification of the effects of chemical agents on living samples. Still, the variance shows the end state of the effect and does not show the instantaneous effect during the process. The variance from when the living samples were adhered to the cantilever and before the chemicals were injected is compared with the variance after the living samples died and detached from the cantilever. Referring to the deflection curve in Figure 6b, it can be noticed that there is damping for the oscillation amplitude of the cantilever that decreases from the highest value, when all the living samples have adhered, to the lowest value, which is equal to the deflection value of the free cantilever without any attached samples. This decrease (damping) takes some time and does not suddenly happen, and damping decreases as the number of adhered samples decreases. Variance does not provide a clear picture of the progression of the reaction of the living samples with chemicals from the beginning of the injection until the samples’ death. Here, we need to find a result display method that enables us to show the instantaneous effect of chemicals on living samples, especially nanoparticles, whose effect may last for several hours or even days.

To overcome drawbacks in displaying the nanomotion results by variance, several methods have been used to display the instantaneous effect of the nanomotion method, as illustrated in Figure 7b–d. The damping coefficient of exponential attenuation that appeared in the cantilever deflection curve was used to define the deflection of the cantilever [127]. The deflection damping coefficient shows the decreasing deflection period from the start of the chemical agents’ effects until the death and detachment of samples from the cantilever surface. Region 3 in Figure 6b shows that the oscillation exhibits exponential reduction, which is defined here by a damping coefficient (B value). As a result, the B value indicates the amplitude of the cantilever, i.e., the damping rate increases as the B value increases. An exponential function was used to represent the oscillation damping, as in the following equation [127]:

(4) $$A\left(T\right)=A_{0}e^{-\mathrm{Bt}}$$

where: *A*_0_ is the amplitude at t = 0. The damping coefficient (B) can then be estimated by solving Equation (4) as:

(5) $$B=-\frac{t}{\tau }\ln\left(\frac{A}{A_{0}}\right)$$

The B value is equal to the negative logarithmic amplitude ratio at any point t in the chemical agents’ effect step and the amplitude at the beginning of the chemical agents’ effect step (t = 0). The B value represents the amplitude damping rate; the higher the B value, the higher the amplitude damping rate. Hence, the more significant the chemical agents’ effect. As shown in Figure 7c, the value of B increases as the concentration of chemical or toxic (in this case) agents rises, and this is valid for various types of chemical agents.

The deflection values of the cantilever were divided into 10-second chunks. Then, a violin plot was plotted for each chunk [158]. The violin plot is a nanomotion spectrogram reconstructed from a histogram. The vertical axis represents cantilever amplitude and the horizontal axis displays the number of oscillation events symmetrically. Figure 7b shows that violin plots were repeated during the different measurement stages. They were used to compare the deflections of the cantilever. The figure shows a change in violin plot height before and after the samples were attached to the cantilever and after the living samples were affected due to the injection of chemical agents. This “violin plot” may be more complex than the variance method but it may enable real-time monitoring of the measurement process.

Power spectral density (PSD) was used to represent the deflections of the cantilever, as shown in Figure 7d. Fourier analysis was used to calculate the PSD of cantilever deflection. The Fourier analysis or the PSD as a nanomotion autocorrelation function may better describe the dynamic response of nanomotion phenomena; this is due to the measurements’ intrinsically stochastic nature. Figure 7d shows the effect of increasing chemical agent concentration on nanomotion deflection compared to the deflection of a cantilever without any chemicals.

### 3.5. Challenges and Future Perspectives

In the studies on nanomotion, several cantilever types have been used, as shown in Table 1. Most of these cantilevers are made of silicon nitrite and coated with a gold layer, some triangular and some rectangular in shape. According to our research, the effects of using cantilevers with different shapes and dimensions or different coating layers on nanomotion deflection when using the same samples and the same experimental conditions have not been studied. The shapes and dimensions of cantilevers have an impact on oscillation specifications and cantilever safety when handling. On the one hand, the gold layer covering the cantilever is crucial to the laser column reflection, which transmits the oscillation deflection of the cantilever to the photodiode. Still, on the other hand, it affects the adhesion of live samples. To ensure the adhesion of living samples on the cantilever surface, cantilevers have been functionalized using different molecules, such as APTES, Glutaraldehyde, Poly-L-lysine, and Poly-D-lysine, as shown in Table 1.

The period in which the measurements were made ranged between ten minutes and seven hours, as shown in Table 1. The time taken for chemical agents to affect living samples varies according to the type of chemical agents and the type of living sample used. Challenge will appear in applications where the chemical agents need a long time to affect the biological samples. Therefore, this will require modifications of the devices used in order to allow real-time monitoring over long periods of up to twenty-four hours or more. Increasing the real-time monitoring period generates other challenges, such as increased noise, increased viscosity of the medium over time, thermal effects, and increasing the time required to process the resulting data.

The AFM oscillating sensor method enables the calculation of the total sample adhesion force by recording the total value of cantilever frequency. The cantilever frequency changes with the number of valid samples still attached to the cantilever surface. The locations of these samples on the surface of the cantilever and away from the free end significantly affect the deflection and frequency of vibrations, but in the AFM oscillating sensor mode, with the final sample, the viability result is not affected by the height of the amplitude but rather by the shape of the drop in amplitude (exponential slope). So, if you assume the localization of samples is neglected in statistical distributions, experiments should be comparable. Thus, we propose that a method be developed in the future which will allow the location of samples on cantilever surfaces to be controlled. This will enable the value of adhesion forces to be calculated. Working on a mathematical model that shows the relationship between adherent samples’ masses and locations on the cantilever surface and the cantilever deflection values may develop the measurement of the viability of a single sample as a focal point for increasing accuracy and reliability.

Nanomotion’s ability to distinguish between living samples based on their propensity to adhere to a surface opens up a wide field of other applications that enable fingerprinting of different living samples according to their health status. Nanomotion was utilized to collect brain tumor vibration signals from cultured cells based on their vibration, allowing for the differentiation of various brain tumors from the normal brain based on nanomotion characteristics [164].

Nanomotion showed significant applicability in the real-time monitoring of the viability of live samples. However, displaying the results so as to enable the real-time monitoring of the effects of chemical agents on living samples still presents a challenge. The result display methods discussed previously are limited either to the end state of the monitoring process or to a specific point during the measurement. Deflection variance, the most commonly used method, gives a visualization of the final state of chemical agents’ effects on living samples but does not show the state of samples instantaneously during the chemical agent step. On the other hand, the deflection damping coefficient value, for example, gives a constant value for the effect on living samples, which may enable a final comparison (depending on the deflection damping curve resulting from the effect of a chemical agent on a living sample) for the state of the living sample at a specific concentration of the chemical agent or comparison between two different chemical agents. Hence, finding a novel display method that enables the display of real-time monitoring of the viability of living samples under the influence of chemical agents may be a promising avenue for future research in this field.

The same living sample and the same chemical agent have not been used in a single study with different result display methods, nor have the different methods been compared. The use of more than one result display method may give a better real-time visualization of chemical agents’ effects on viability as measured by nanomotion. A time-dependent study (amplitude modulation (AM) and frequency modulation (FM) over time) was used to show the single-cell force spectroscopy measure of yeast cell metabolism [166]. For all the result display methods, the focus has been on amplitude modulation characters but frequency modulation has not been analyzed for results in order to determine the characteristics of frequencies at the different stages of the measurement process, which information could have revealed a new horizon for the visualization of the results of the viability measurement process using nanomotion. Nanomotion oscillation signals can be converted into sound signals within the human hearing frequency band [164], providing novel concepts that could lead to the development of intelligent detection devices for real-time viability measurement.

## 4. Conclusions

In this review, the most common methods used for living sample viability measurement have been classified and presented, and their benefits and drawbacks, as well as potential utilities, have been discussed and evaluated. The principles and features of VMMs are summarized in Table 3. Chemical assays are the most widely used method for measuring cell viability. They can be used if the goal is to know the endpoint of a cell’s fate. Optical methods measure the viability of living samples by monitoring and imaging morphological changes of the samples, and the efficiency of database processing via deep learning software limits their accuracy and scope. All of the studies in the literature discussed here certify that nanomotion is a promising alternative tool for measuring the viability of living samples which effectively achieves rapid, quantitative, direct, and real-time determination. Nanomotion has been used to study biological activities, toxicity, and drug efficacy in living samples, such as bacteria, cells, sperm, and cellular organs, such as mitochondria and ATP. Sample attachment protocols and result display methods are the critical factors in the progress of VMMs. Further development in these two processes is essential to the enhancement of the efficiency and repeatability of results and the enabling of instantaneous monitoring. To avoid different environmental conditions during the measurement process, sample adhesion to a cantilever surface can be achieved by means of a direct attachment protocol outside the test room or by injecting the sample into the test room to avoid different environmental conditions and reduce the possibility of contamination or cantilever damage. If needed, more complex protocols, such as the micrometric motors of the AFM or ink-jet printing, can be employed to control the numbers and positions of samples on the cantilever surfaces. The present nanomotion result display methods do not show the actions of agents on live samples changing instantly in real-time.

Additionally, cantilever type and functionalization are important factors that affect the success of nanomotion methods, while the numbers and positions of adherent samples on cantilevers are significant factors affecting the reliability and repeatability of the process. We sincerely hope that this review will drive the future development of nanomotion and provide significant thoughts for novel VMMs.

## Figures and Tables

**Table 2:** AFM nonomotion living sample attachment protocols.

Attachment Protocol	Incubation Condition	Advantages	Drawbacks	Ref.
**Cantilever incubated in sample medium outside of the AFM test room**	The adhesion process is carried out under different conditions of the chemical effect process	Easy and no need for expensive equipment	The location and number of cells or bacteria cannot be controlled; When handling and installing the cantilever, there is a risk of contamination, sample death, or cantilever damage	[143,148,149,151,153,154,155,157,158,159]
**Inject sample medium inside the test room**	The adhesion and chemical effect processes are carried out in the same test room and under the same conditions	All measurement processes are carried out under the same conditions; There is no risk of contamination or death of cells or bacteria	The location and number of cells or bacteria cannot be controlled; Requires high sample concentration	[126,127,150,156]
**The micrometric motors of the AFM** **—** **AFM single-cell force spectroscopy**	The adhesion and chemical effect processes are carried out in the same test room and under the same conditions	The location and number of cells or bacteria can be controlled; It is a single-cell and multi-cell measurement process	Complex and expensive equipment; There is a risk of cell injury during the adhesion process; A sample is limited by its size and by cantilever size	[120,151,152]
**Ink-jet printing**	The adhesion and chemical effect processes are carried out in the same test room and under the same conditions	The location of cells or bacteria can be controlled; There is no risk of contamination or death of cells or bacteria	Complex and expensive equipment is needed; The number of cells or bacteria cannot be controlled	[161,165]

**Table 3:** Principles and features of VMMs.

Measurement Method	Principle	Features
**Chemical viability assays**	Injection of chemical compound(s) into living samples and evaluation of sample interaction with these compound(s)	Labelled and multi-sample methods Easy, inexpensive, and no need for complex techniques Suitable for either suspended or adherent samples Assay identification and design depend on the drug’s nature and the type of biomarkers used Endpoint assays For a large number of samples, it is time-consuming and labor-intensive
**Raman spectroscopy**	Detection of morphological changes	Rapid, label-free, contactless, and multi-sample method Real-time method, non-invasive and not damaging to samples RS results are affected by the weak Raman signal and light scattering, which reduce the device’s sensitivity Time-consuming and human factor errors for a large number of samples Machine-learning algorithms must be used for high-throughput screening
**Flow imaging microscopy**	Detection of morphological changes of living samples while the sample fluid is in a continuous flow	Rapid, label-free, contactless, and multi-sample method Real-time method, non-invasive and not damaging to samples High throughput Able to measure samples one by one and numerically calculate size distribution using a convolutional neural network with deep learning technology Able to solving critical sample classification problems through conjunction with image-processing technology and advanced machine-learning algorithms The speed of data analysis is the most significant limitation
**Holography**	Detection of rapid changes in living sample structure parameters resulting from mechanical or morphological changes	Rapid, label-free, contactless, and multi-sample method Real-time method, non-invasive and not damaging to samples Suitable for direct observation of 3D bio-tissue without scanning Accuracy is affected by light scattering and light source quality
**On-chip, lensless video microscopy technology**	Detection and evaluation of changes in the shadows of living samples	Rapid, label-free, contactless, and multi-sample method Real-time method, non-invasive and not damaging to samples Has twice the visual field of a conventional microscope No requirements for optical or mechanical elements, such as lenses or probes By using microfluidic channels, it is possible to monitor more than one living sample type simultaneously Machine-learning algorithms must be used for high-throughput screening Possibility of phototoxicity
**Respiratory measuring methods**	Detection of the oxygen absorbed and consumed by a living sample	Rapid, label-free, contactless, and multi-sample method Real-time method, non-invasive and not damaging to the samples Continuous high-throughput method Sensitive to environmental parameters, such as temperature, pressure, flow, and salinity Calibration difficulty, poisoning risk, oxygen consumption, and high costs, especially for large samples Sensor materials need to have low oxygen permeability and easy-to-manufacture thermoplastic polymers
**Microcalorimeter measuring methods**	Detection of the resulting heat from a living sample	Rapid, label-free, contactless, and multi-sample method Real-time method, non-invasive and not damaging to samples Continuous high-throughput method Sensitive to environmental parameters, such as temperature, pressure, flow, and salinity The complexity of results interpretation and accuracy affected by radiation absorption Sensor resolution is not accurate enough to match the single-sample temperature, measured in pW Calculating the average heat generated by colonies of living samples
**Nanomotion**	Take advantage of the AFM cantilever’s high sensitivity to changes in mass caused by sample adherence to the cantilever surface	Rapid, label-free method Real-time method, non-invasive and not damaging to the samples Applicable to either single or multiple samples Measurement time is reduced to several hours instead of several days, as with traditional assays Able to monitor the instantaneous effects of chemical agents on living samples for several hours or even days Unlike single-cell force spectroscopy, adhesion is evaluated without forcing the living sample to detach through cliffs or stretching Cantilever surface functionalization is needed for sample attachment The current nanomotion result display methods do not show the instantaneous effects of chemical agents on living samples
